# Supplementary material for: A whole-animal phenotypic drug screen identifies suppressors of atherogenic lipoproteins
Source: eLife. 2026 Aug 27;14:RP105314. doi: 10.7554/eLife.105314 (PMC13521566; doi:10.7554/eLife.105314)
Supplement: Supplementary file 2 [file elife-105314-code1.zip › JHDL_validation_result/Nabumetone_UK20210719_02.pdf]

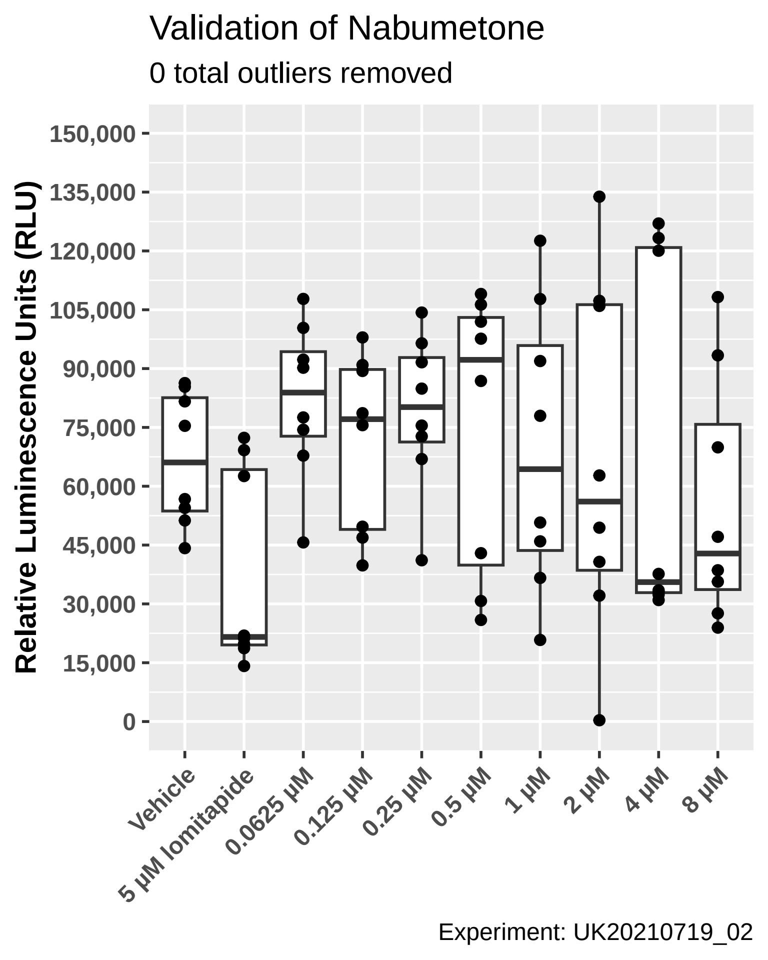

Validation of Nabumetone
0 total outliers removed
Relative Luminescence Units (RLU)
150,000
135,000
120,000
105,000
90,000
75,000
60,000
45,000
30,000
15,000
0
Vehicle
5 μM lomitapide
0.0625 μM
0.125 μM
0.25 μM
0.5 μM
1 μM
2 μM
4 μM
8 μM
Experiment: UK20210719_02
